# Triple Benefit: Impact of an Integrated Early Childhood Development and PMTCT Intervention on ART Outcomes Among Mothers Living with HIV and Infants in Malawi—An Endline Evaluation

**DOI:** 10.1007/s10461-022-03975-0

**Published:** 2023-02-06

**Authors:** Kathryn Dovel, Pericles Kalande, Evelyn Udedi, Laurie Bruns, Chris Desmond, Chipariro Mbalanga, Sundeep Gupta, Sam Phiri, Mackenzie Chivwala, Linda Richter, Thomas J. Coates

**Affiliations:** 1 Partners in Hope, Implementation Science, Lilongwe, Malawi; 2 Division of Infectious Diseases, David Geffen School of Medicine, University of California Los Angeles, 10833 Le Conte Ave, Los Angeles, CA 90095 USA; 3 University of California Global Health Institute, San Francisco, CA USA; 4 Stellenbosch University, Stellenbosch, South Africa; 5 University of the Witwatersrand, Johannesburg, South Africa

**Keywords:** Child development, HIV, Maternal-Child Health Services, Delivery of Health Care, Integrated, Sub-Saharan Africa, Malawi

## Abstract

We conducted a programmatic, observational cohort study with mother-infant pairs (MIPs) enrolled in prevention-of-mother-to-child-transmission (PMTCT) programs in Malawi to assess the feasibility and potential HIV-related benefits of integrating Early Childhood Development (ECD) services into PMTCT programs. Six health facilities were included in the intervention. We offered ECD counseling from the WHO/UNICEF Care for Child Development package in PMTCT waiting spaces while MIPs waited for PMTCT and broader treatment consultations. Primary outcomes were mothers’ retention in HIV care at 12 months and infant HIV testing at 6 weeks and 12 months after birth. Routine facility-level data from six comparison health facilities were collected as an adhoc standard of care comparison and used to calculate the cost of delivering the intervention. A total of 607 MIPs were enrolled in the integrated ECD-PMTCT intervention between June 2018 and December 2019. The average age of MIPs was 30 years and 7 weeks respectively. We found that 86% of mothers attended ≥ 5 of the 8 ECD sessions over the course of 12 months; 88% of intervention mothers were retained in PMTCT versus 59% of mothers in comparison health facilities, and 96% of intervention infants were tested for HIV by six weeks compared to 66% of infants in comparison health facilities. Costing data demonstrated the financial feasibility of integrating ECD and PMTCT programs in government health facilities in Malawi. Integrating ECD into PMTCT programs was feasible, acceptable, resulted in better programmatic outcomes for both mothers and infants. Further investigation is required to determine optimal delivery design for scale-up.

## Introduction

Prevention of mother-to-child-transmission (PMTCT) programs in sub-Saharan Africa have progressed remarkably, with many countries reaching 90–95% treatment coverage among pregnant women living with HIV and reducing vertical transmission to children [1]. However, retention in PMTCT programs and adherence to antiretroviral treatment (ART) remains a challenge in the postpartum period [2–4]. The higheset losses in retention occur postpartum since mothers no longer attend health facilities for routine antental and postnatal visits—PMTCT is the only reason to attend facilities frequently. A cohort study from South Africa found that 35% of postpartum women defaulted from ART services within 6-months postdelivery, and nearly 50% missed at least one scheduled ART appointment during that same period [5].

Barriers to postpartum women’s retention in PMTCT programs include fear of HIV stigma, lack of social support, feeling healthy (i.e. no immediate benefit of treatment), treatment fatigue after taking ART throughout pregnancy, and financial constraints for monthly PMTCT visits postpartum [6, 7]. New motherhood can exacerbate barriers due to increased familial responsibility, decreased time availability, and potential postpartum depression [8, 9]. In addition, frequent facility visits required for PMTCT services may be particularly challenging postpartum. In Malawi, postpartum women are expected to attend the health facility every month for the first six months postpartum for PMTCT services, including mother’s ART dispensing and monitoring of the infant [10]. With an average four-hour wait-time for ART services [11], monthly facility visits solely for PMTCT require substancial time and resources that may clash with women’s increased familial responsibilities and constrained household budgets. Disrespectful or negative client-provider interactions may be especially demotivating in light of the above barriers [12, 13]. Novel, scalable strategies are needed to mitigate the burden of PMTCT services on postpartum women, with the ultimate goal to improve PMTCT retention and child outcomes.

Integrated, holistic services may improve client experiences at PMTCT visits and increase the benefit of mothers’ monthly PMTCT appointments. Integrated interventions promise to maximize clinic resources, improve health system efficiency, better utilize client time spent at health facilities, and improve client satisfaction since clients may feel their time at the facility was well spent [14]. Early childhood development may be one form of integrated services that improves mothers’ experiences with PMTCT and simultaneously promote children’s overall wellbeing.

Early childhood development interventions aim to support parents and children to ensure that children reach their developmental potential, with most interventions targeting mothers of young infants [15]. The WHO/UNICEF Care for Child Development (CCD) curriculum [16] provides parental support to increase responsive caregiving through basic ECD parenting trainings that usually include group education, peer support, and role play with children on how to promote development through responsive caregiving.

We integrated ECD activities, using the WHO/UNICEF CCD curriculum, into existing PMTCT services and delivered ECD sessions to postpartum mothers while they waited for routine PMTCT consultations. We conducted a formative assessment on feasibility and acceptability and found that an integrated ECD-PMTCT program provided additional motivation for PMTCT attendance because mothers were strongly motivated to attend ECD sessions to improve their children’s developmental outcomes [12]. Mothers were also less frustrated with long wait times for PMTCT services since they received ECD activities during these times, and also gained social support from other mothers and health care workers (HCWs) during ECD sessions. In this paper, we report the impact of the integrated ECD-PMTCT intervention on HIV-related outcomes for mother and infant.

## Methods

### Study Design and Participants

**Fig. 1:**
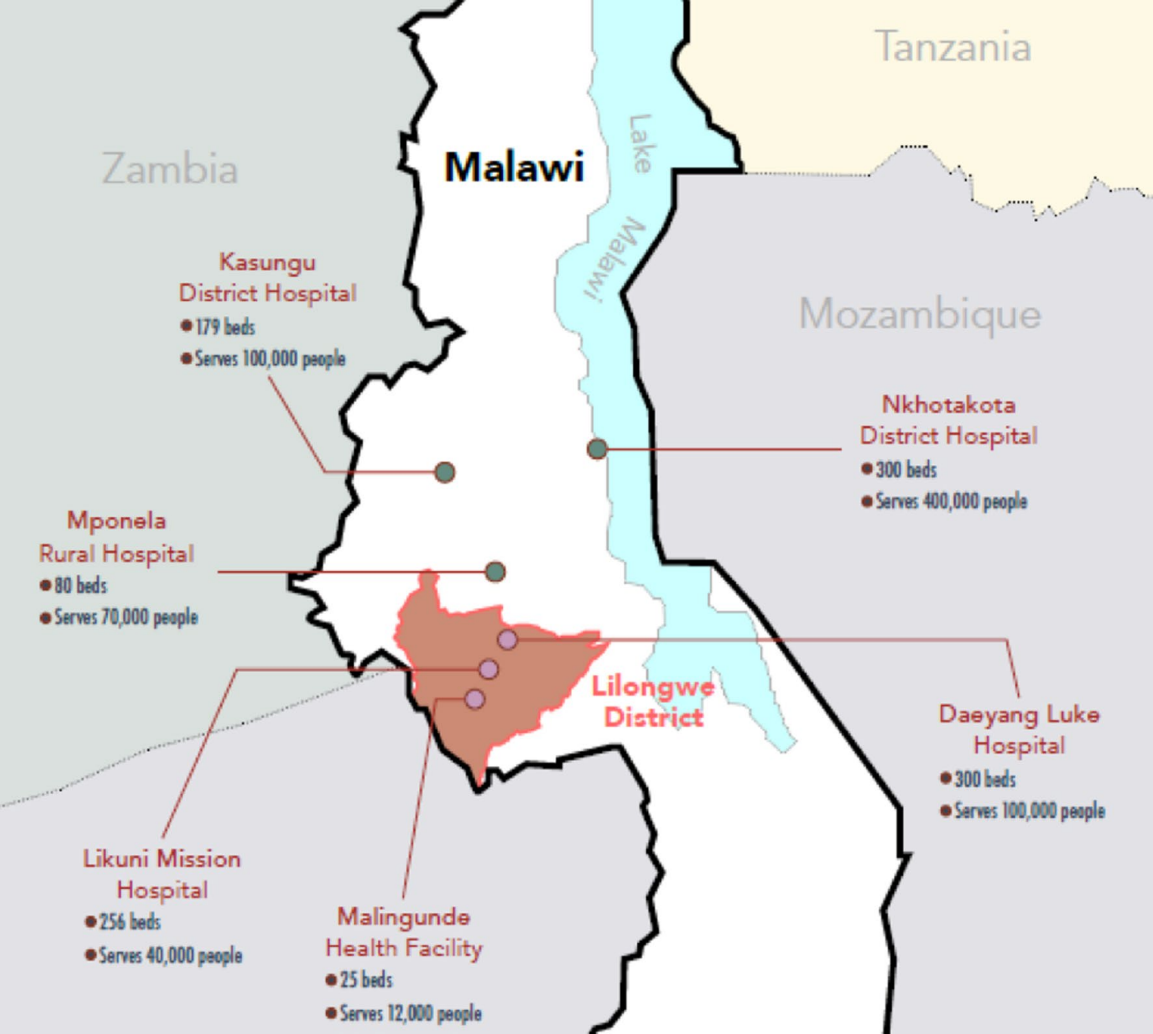
Map of study sites in Central Malawi

We conducted a programmatic, observational cohort study to understand women’s engagement in the integrated ECD-PMTCT intervention and HIV-related outcomes among mother-infant pairs (MIPs). Primary outcomes of interest include ART retention among women 12 months after enrollment in the intervention (defined as < 28-days late for their most recent ART appointment) and HIV testing for infants at six weeks and 12 months of age. Six health facilities in central Malawi were purposively selected to participate in the study as intervention sites. Selected sites had to (1) have an active PMTCT program with a separate PMTCT clinic; (2) be located in central Malawi; and (3) be supported by Partners in Hope, a PEPFAR implementing partner that supports HIV service delivery in over 100 facilities in Malawi. We purposively selected facilities that met these criteria in order to have a range of sites across districts and facility types. The selected sites spanned a range of large public facilities (n = 3), and mission hospitals (n = 3) (see Fig. 1). All facilities offered separate PMTCT clinics where MIPs could receive HIV-related services (ART services for mothers, HIV testing and treatment, if needed, for infants). Additional services such as family planning, care for non-communicable diseases (NCDs), and acute care for other illnesses were not integrated into the services.

Women were eligible for the study if they were enrolled in a PMTCT program at one of the participating health facilities, mothers were 18 years of age or older at the time of enrollment, and their youngest child was aged six weeks old at the time of recruitment.

### Intervention

**Fig. 2:**
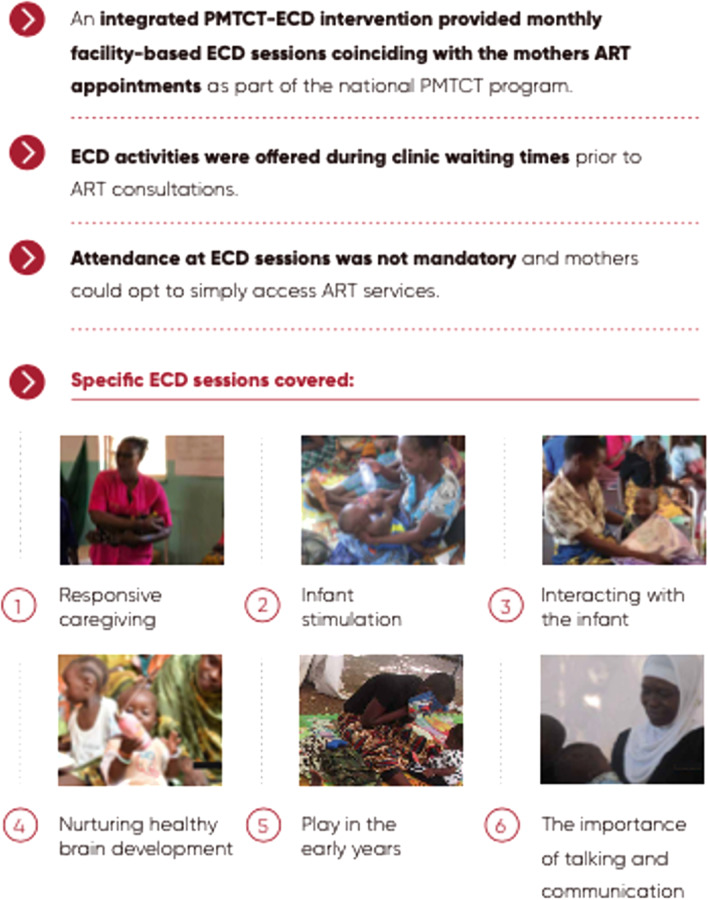
Study protocols for intervention and data collection

A combined ECD-PMTCT intervention was integrated into the national PMTCT program for postpartum women and their children (see Fig. 2). The national standard PMTCT program includes monthly ART clinic visits for 6 months postpartum, followed by 2, 3 or 6 month visits thereafter depending on treatment adherence, usually measured by ART pill count. Mothers were monitored for their adherence to their ART regimen during each clinic visit, received their ART refills, and were counselled on ART adherence and on the importance of exclusive breastfeeding. Infants received routine dry blood spot (DBS) tests at six weeks and routine rapid tests, blood-based Determine 1/2 (Abbott Laboratories; Chicago, IL, USA) at 12 months. A positive test is confirmed using Uni-Gold (Trinity Biotech; Bray, Ireland).

The ECD-PMTCT intervention provided facility-based ECD sessions coinciding with mothers’ routine PMTCT clinic appointments. We used the WHO/UNICEF CCD package [16] and focused on the cognitive, emotional and communication development of infants, as well as promoting caregiving sensitivity and responsivity of mothers through support and play. The integrated ECD-PMTCT intervention was implemented by Expert Clients, a community-based, lay cadre of HCWs who are living with HIV and had successfully achieved ART adherence.

Mothers were encouraged to attend group ECD sessions while waiting to receive routine PMTCT services. Group sessions engage 4 to 10 MIPs waiting for PMTCT services, allowing mothers to learn from each other and develop social support networks given that many mothers attend repeat PMTCT services on the same day due to similar ART refill schedules. Attendance at ECD sessions was voluntary. Mothers have the opportunity to attend approximately eight ECD sessions as part of routine PMTCT appointments over the course of 12 months. If desired, mothers could also opt to attend additional ECD sessions on non-PMTCT appointment days.

### Procedures

Eligibility criteria included: being enrolled in a PMTCT program at one of the participating health facilities; youngest child aged 0–8 weeks at the time of recruitment, and mothers ≥ 18 years of age at the time of recruitment. Postpartum mothers attending PMTCT services were approached while waiting for routine care (often in the waiting rooms outside the PMTCT-specific ART clinic) and screened for study eligibility in a private, quiet location within the health facility. Study staff attempted to recruit all mother–infant pairs presenting for PMTCT. However, due to high volumes of clients not all mothers were recruited. Eligible MIPs completed informed consent and were offered the ECD-PMTCT intervention either immediately after completing consent (if enrolled prior to receiving PMTCT services for that day) or at their next monthly PMTCT visit (if enrolled after PMTCT services were completed). Enrollment took place on a rolling basis, with as many MIPs enrolled as eligible at each facility. As a result, the number of study participants per facility varied and was based on the size of the PMTCT cohort within each facility.

A brief baseline survey was completed immediately after enrollment to capture mothers’ demographic variables, ART initiation date and parity (number of children and age of youngest child). Integrated ECD-PMTCT medical registers were kept by HCWs and updated in real time whenever MIPs attended PMTCT clinics. Register data included the following information: whether ECD service was offered; if yes, ECD topics covered, and HIV services received (ART distribution and/or recording adverse events for mothers; HIV testing and results for infants). At the end of the follow up, ECD-PMTCT medical registers were digitized and 12-month outcome data ascertained, along with MIPs exposure to ECD sessions throughout the 12 months post-enrollment.

As part of a sensitivity analysis to compare 12-month outcomes for the integrated ECD-PMTCT intervention against standard of care PMTCT, we purposively selected six comparable health facilities posthoc (after the ECD intervention was completed). The comparison sites had the same eligibility criteria as intervention sites (have an active PMTCT program with a distinct PMTCT clinic; located in central Malawi; and be supported by Partners in Hope), but did not implement any ECD activities during the study timeframe. We purposively selected from sites eligible to be comparison facilities by matching sites with study ECD facilities based on facility type, district, and size of the PMTCT program (see Table 1). Facility-level data were collected on the same 12 month PMTCT outcomes for MIPs between the same timeframe as endline data collection for the intervention sites (June 2019 and December 2020).

**Table 1:** Characteristics of study facilities and comparison facilities

Facility characteristic	Treatment site	Comparison site
	(n = 6)	(n = 6)
District		
Dowa	1	1
Lilongwe	3	3
Nkhotakota	1	1
Kasungu	1	1
Facility type		
Mission	3	3
Public	3	3
Facility size		
Health Centre	1	1
Hospital	3	3
District Hospital	2	2

### Outcomes and Statistical Analysis

We analyzed data from MIPs who reached 12 months after study enrollment (including those lost-to-follow-up, as long as they had been enrolled in the study > 12 months prior). The primary outcome of interest was mothers’ ART retention 12 months after study enrollment. Retention was defined as having no adverse event reported (including defaulted, defined as > 60 days late for ART appointment, transferred out, or died). Secondary outcomes included infant’s routine HIV testing using DBS tests at six weeks and rapid, blood based Determine 1/2 at 12 months. Process evaluation outcomes included number of ECD sessions attended by MIPs during the 12 month study enrollment and missed ECD opportunities during the same time period (defined as MIPs who attended PMTCT services but not ECD sessions on that same day).

We used CONSORT standards for reporting trial outcomes [17]. Descriptive statistics (mean, SD, median, IQR, and frequency distribution) were conducted to describe baseline characteristics of participants, their engagement in the ECD-PMTCT intervention, and PMTCT-related outcomes at 12 months after study enrollment. We used intention-to-treat principles for the primary outcome analysis (mothers’ retention in ART services at 12 months), whereby MIPs who were lost-to-follow-up or had missing primary outcome data were considered as outcome failures.

### Cost Data

We estimated the financial cost to providers for delivering the intervention in an independent analysis. Monitoring systems, including timesheets, staff interviews and expenditure categorization were established from the outset to distinguish between research and implementation costs. An ingredients approach was used to estimate the implementation cost per activity. These data were then used to calibrate a costing model to estimate the likely costs if the intervention is replicated. We report three replication scenarios: (1) intervention delivered by the government through a ministry of health clinic; (2) intervention delivered by the government through a ministry of health clinic but with lower levels of supervision; and (3) intervention delivered by a non-governmental organization (NGO). To provide a measure of relative efficiency, we report the cost per ECD session. ECD sessions are the entry point for the intervention and therefore provide a measure of the scale of intervention. The scale gets larger as more sessions are added. The cost implications of expanding integrated ECD-PMTCT services is determined by the structure of health care delivery: what level of existing cadre can implement the service, how much they are paid, and the level and cost of supervision they require. The scale of implementation is the same in Scenarios 1 and 2, but Scenario 2 estimates the cost implications of less supervision. Scenario 3 involves staff with higher skill levels and increased supervision.

### Ethical Approval

Ethical approval was attained from University of California Los Angeles (UCLA) and the Malawi Institutional Review Board, National Health Sciences Research Committee (NHSR).

## Results

Approximately 85% of MIPs with newly born infants attending PMTCT services were screened and eligible for the study. The primary reason for non-enrollment was not being reached by study staff during busy clinic sessions. Fewer than 2% of mothers refused participation. A total of 607 MIPs were enrolled in the integrated ECD-PMTCT intervention between June 2018 and December 2019, and endline data were collected between June 2019 and December 2020.

The mean age of mothers at enrollment was 30 years (SD: 7 years) while infants had a mean age of 7 weeks (SD: 12 week) (see Table 2). At the time of enrollment, mothers had been on ART for a mean 39 months, with high levels of variability (SD: 38 weeks). About two-thirds (61%) of MIPs were enrolled at public health facilities, 39% were enrolled at mission hospitals. The district hospital contributed 35% of MIPs enrolled.

**Table 2:** Characteristics of women enrolled in the integrated ECD-PMTCT at least 12 months prior to data collection (n = 607)

Variables	% (n)
Demographics and ART services	
Mother’s age (in years), mean (SD)	30.45 (6.49)
Months on ART (since ART initiation), mean (SD)	39.32 (37.95)
Infant’s age (in weeks), mean (SD)	7.1 (11.5)
Facility details	
Facility type	
Public Facilities	61.45 (373)
Mission Hospitals	38.55 (234)
Facility	
Facility 1: Mission Hospital	18.45 (112)
Facility 2: District Hospital	34.60 (210)
Facility 3: Mission Hospital	11.04 (67)
Facility 4: Health Centre	9.06 (55)
Facility 5: Rural Hospital	6.26 (38)
Facility 6: District Hospital	20.59 (125)

**Fig. 3:**
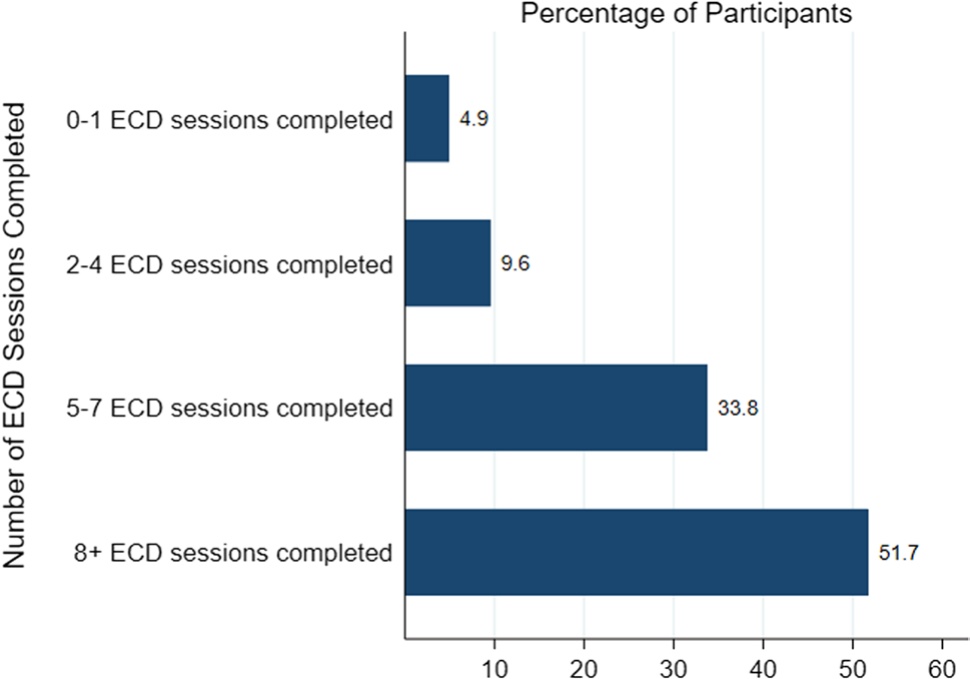
ECD sessions attended over 12 months of enrollment (n = 607)

**Fig. 4:**
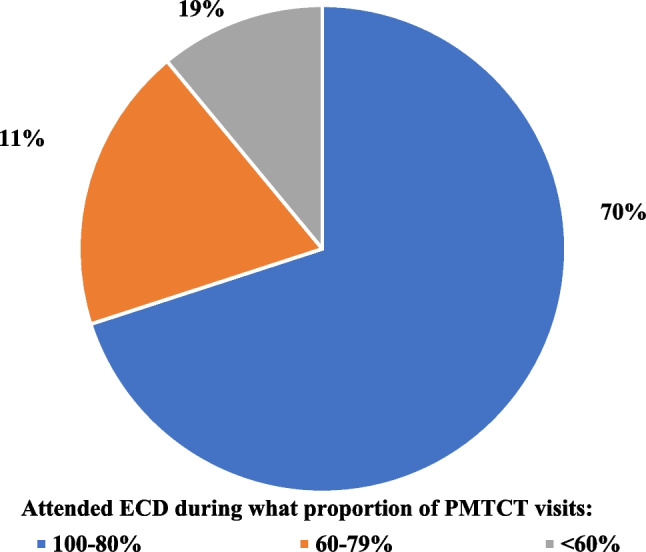
Proportion of MIPs who attend an ECD session during their routine PMTCT facility visits within the 12 months after enrollment (n = 607)

Figure 3 shows the number of ECD sessions attended by MIPs over the 12-month study period. Out of the 607 MIPs enrolled, the majority (86%) attended 5 or more ECD sessions while 52% attended 8 or more ECD sessions. Figure 4 shows the proportion of concurrent PMTCT and ECD visits made by MIPS; 70% of women attended an ECD session during at least 80% of their routine PMTCT facility visits, showing high coverage and few missed opportunities. The primary reason for missing ECD sessions was that ECD services were not offered when women attended PMTCT services (usually very late in the day, outside peak hours), while a minority of women did not attend because ECD sessions required additional time (in cases where there was no wait time to access PMTCT services).

Table 3 shows PMTCT-related outcomes at 12 months. Of the 607 MIPs enrolled, 88% were retained in the PMTCT program at 12 months. Only 4% defaulted (> 60 days late for any ART appointment), 7% transferred to other facilities for their PMTCT care, and six women died in the 12-month period (1%). Rates of default were similar across mothers’ age and facility attended (not shown). Among infants, 96% (581) had a DBS test conducted at six weeks after birth, with 2% testing HIV-positive; 80% of infants had an HIV test taken at 12 months (using blood-based tests) and 1% of tests were reactive.

**Table 3:** 12-month PMTCT-related outcomes among women enrolled in an integrated ECD-PMTCT program in Malawi (n = 607)

Variables	% (n)
Retained in the PMTCT program	87.97 (534)
Mother-Infant Pair Adverse Events	
Transferred	7.25 (44)
Died	0.99 (6)
Default (> 60 days late for an ART appointment)	3.79 (23)
Infant HIV-testing	
6-week test	95.72 (581)
Positivity rate	1.92 (10)
12-month test	79.57 (483)
Positivity rate	1.09 (5)

Data from the six comparison facilities shows poorer PMTCT outcomes as compared to study participants. In comparison sites, only 59% of mothers were retained in care 12 months after birth (with 18% default); only 66% of infants were tested for HIV using DBS less at six weeks and 78% of infants received HIV testing at 12 months.

### Cost of Intervention

 The activities per month, the staff required to implement them, and the associated cost are reported for each scenario (see Table 4). The cost per integrated ECD session ranged from US$10 (delivered at government clinics) to US$28 (delivered at NGO run clinic). The cost is higher at the NGO clinic because it also includes a higher wage for the delivery and supervisory staff and more intensive supervision. The costs at government clinics assume implementation by existing health care workers, as well as lower levels of supervision, both to which substantially reduce costs.

**Table 4:** Monthly activity level, staff requirements and cost (USD), by scenario

	Scenario 1^†^	Scenario 2^‡^	Scenario 3^§^
Activities per month			
General health talks conducted	15,485	15,485	7520
Pamphlets distributed	32,796	32,796	18,063
Clients screened	34,515	34,515	17,820
Clients enrolled	3734	3734	2790
Individual ECD sessions conducted	10,650	10,650	5685
Group ECD sessions	6721	6721	5022
Staffing requirements (FTE)			
Expert clients (lay cadre)	715	715	258
Supervisors	29	15	30
Managers	8	4	8
Senior managers/administrators	8	4	8
Support staff	24	12	24
Monthly cost (USD)			
Staff	117,166	109,451	180,653
Program costs	13,871	13,871	5005
Transport/travel costs	11,710	6057	12,114
Communication	1191	616	1233
Meeting costs	850	440	879
Office costs	7229	3739	7478
Overhead	5310	2747	5493
Total cost per month	157,328	136,920	212,855
Annual cost	1,887,936	1,643,040	2,554,263
Cost per ECD session (relative efficiency)	10	9	28

^**†**^Scenario 1: Intervention delivered by the government through Ministry of Health without ECD specific supervision

^‡^Scenario 2: Intervention delivered by the government through Ministry of Health with low level supervision on ECD activities

^§^Scenario 3: Intervention delivered by an NGO with dedicated lay cadre for intervention implementation

## Discussion

We found that an integrated ECD-PMTCT intervention implemented in Malawi in facility waiting spaces is feasible and has important PMTCT-related outcomes. The intervention was highly acceptable; 86% of mothers attended the majority of ECD sessions (which were all optional and not tied to receiving PMTCT services). We found that 88% of postpartum mothers were retained in ART services at 12 months (with only 4% defaulted) and 96% and 80% of infants received HIV testing at 6 weeks and 12 months, respectively. Using aggregate facility-level data from our six comparison facilities, we find that standard of care outcomes from comparable facilities show only 59% of postpartum mothers were retained in care 12 months after birth (with 18% defaulted) and only 66% and 78% of infants were tested for HIV using DBS at 6 weeks and 12 months.

Postpartum mothers and their infants in the integrated ECD-PMTCT program showed promising, sustained engagement in PMTCT services, with very low rates of default by 12 months postpartum. While our analysis cannot explain why retention is higher in our study, a strong hypothesis is that it is due to the integration of ECD sessions. First, the integrated ECD sessions provide repeat interactions with groups of mothers living with HIV, potentially improving social support. Other literature shows social support, either by ‘expert mothers’ or fellow mothers, to be a critical component of ART adherence amongst this population [18–20]. Second, qualitative data show that mothers perceive client-provider interactions to improve due to increased interaction within the integrated ECD-PMTCT program. In PMTCT waiting spaces mothers have in-depth conversations with providers regarding family and parenting, and mothers have a sense of belonging within the health facility—humanizing the facility experience [21, 22]. Positive client–provider interaction is a known facilitator to ART engagement within the context of PMTCT programs in sub-Saharan Africa [23]. Finally, the introduction of the ECD curriculum may have provided additional motivation to attend health facilities on a regular basis as mothers are highly motivated by their wellbeing of their children and learning new skills to promote child wellbeing.

Several limitations should be noted. First, we did not conduct a randomized controlled trial and the adhoc comparison facilities may be qualitatively different from intervention facilities, even though we matched comparison and intervention facilities on key facility characteristics. Nonetheless, our findings are encouraging and show substantial improvement on retention within routine PMTCT programs [2]. Second, additional work is needed to understand health care worker perspectives on the scalability and sustainability of integrated ECD-PMTCT services. Identifying key facility- and health system-level strategies to promote ongoing integration and quality implementation will be critical to achieving similar outcomes in routine care settings.

Integrated ECD-PMTCT services are affordable in a clinical setting. However, further investigation is required to determine optimal delivery design while retaining effectiveness at scale. The cost implications of expanding access to integrated ECD-PMTCT services will be determined by how much implementing staff are paid and the level of supervision they receive, as well as the salaries supervisors are paid. Lower wages could lower the cost significantly, as would lower levels of supervision, but both might compromise quality. A further key consideration is the extent to which the intervention can be integrated, particularly in terms of the use of existing facility staff. If implementing staff can work on other projects while not busy with the ECD intervention, the intervention becomes more efficient.

## Conclusion

ECD integrated into PMTCT programs was feasible, acceptable, and resulted in better PMTCT programmatic outcomes for both mothers and infants. Further investigation is required to determine optimal strategies for scale-up.

## Data Availability

Data are available upon request from the lead authors.

## Abbreviations

ART: Antiretroviral treatment
CCD: WHO/UNICEF Care for Child Development
DBS: Dry blood spot
ECD: Early Childhood Development
HCW: Healthcare worker
MIP: Mother–infant pair
NCD: Non-communicable disease
NGO: Non-governmental Organization
PMTCT: Prevention-of-mother-to-child-transmission

## Code Availability

N/A.
